# Underlying Causes and Co-existence of Malnutrition and Infections: An Exceedingly Common Death Risk in Cancer

**DOI:** 10.3389/fnut.2022.814095

**Published:** 2022-02-23

**Authors:** Yuanyuan Fan, Qianqian Yao, Yufeng Liu, Tiantian Jia, Junjuan Zhang, Enshe Jiang

**Affiliations:** ^1^School of Life Sciences, Henan University, Kaifeng, China; ^2^Institute of Nursing and Health, Henan University, Kaifeng, China; ^3^DeDepartment of Orthopedics, Henan Provincial People's Hospital, Zhengzhou, China; ^4^Henan International Joint Laboratory for Nuclear Protein Regulation, Henan University, Kaifeng, China

**Keywords:** malnutrition, infections, children under five, cancer, developing countries, future strategies

## Abstract

In nutrition science, malnutrition is a state of imbalance between intake and the needs of the organism, leading to metabolic changes, impaired physiological functions, and weight loss. Regardless of the countless efforts being taken and researched for years, the burden of malnutrition is still alarming and considered a significant agent of mortality across the globe. Around 45% of 12 million children deaths (0–5 years old) annually are due to malnutrition, mostly from developing countries. Malnutrition develops associations with other infections and leads to substantial clinical outcomes, such as mortality, more visits to hospitals, poor quality of life and physical frailty, and socioeconomic issues. Here, in this review, we intend to provide an overview of the current burden, underlying risk factors, and co-existence of malnutrition and other infections, such as cancer. Following the rising concern of the vicious interplay of malnutrition and other medical illnesses, we believed that this narrative review would highlight the need to re-make and re-define the future strategies by giving comprehensive and sustainable programs to alleviate poverty and combat the rampant infectious diseases and those nutrition-related health problems. Furthermore, the study also raises the concern for hospitalized malnourished cancer patients as it is crucially important to knowledge the caregiver healthcare staff for early interventions of providing nutritional support to delay or prevent the onset of malnutrition.

## Introduction

For in nutrition sciences, malnutrition is the state of the disproportion of food intake and energy requirement of an organism, which leads to impaired metabolism, body dysfunction, and diminishing of body mass (1, 2). Malnutrition is considered as a multifactorial and diverse ailment of all ages. It has been reported worldwide and has gained substantial significance as a leading health issue of the current time. Statistically, literature revealed that about 12 million children (aged 0–5years) die every year and the major portion of these deaths is malnutrition (3). Findings reveal that malnutrition is the major contributor to disease burden in developing countries where one out of every three preschool-age children were affected by malnutrition (4–6). Sub-Saharan Africa is experiencing the highest burden of malnutrition with the high level of undernutrition and growing burden of overweight/obesity and diet-related non-communicable diseases (7). It is estimated that around 1.9 billion adults are overweight while more than 462 million adults are underweight (8). According to a study supported by Bill and Melinda Gates Foundation, malnutrition is the predominant risk factor for death in children younger than 5 years of age in every state of India (9). The results of prevalence studies demonstrate the worse look of malnutrition in hospitals where 50% of patients were found malnourished at admission and more patients may be malnourished upon the hospital discharge (10, 11). In health-related outcomes, malnutrition affects individual physical growth, mobility, cognitive development, and extended length of hospital stay (12), institutionalization (11), poor quality of life (13), and physical frailty in hospitalized patients (14). Malnutrition develops a vicious association with other diseases, such as diarrhea, measles, cancer, and HIV, that lead to increased malnutrition-related death that further imposes both manpower and economic loss epically in poor countries (15–17).

Presently, malnutrition is considered as a significant agent of mortality across the globe, and it is urgently needed to design the strategies to cope with the challenges about the prevention of malnutrition: common nomenclature, screening methods, accurately reported data, and defined distinct silos to address the malnutrition by bridging the existing gaps (18–21). The current narrative review aimed to summarize the literature and provide up to date on underlying causes, characteristics, and the prevalence of malnutrition. With more emphasis, the review highlights the complex interactions and co-existence of malnutrition and other diseases, which govern a vicious cycle to end life especially in cancer-related malnutrition.

## Method and Literature Mining Strategy

Relevant articles were selected against the specific keywords as per outlines of study by using the different search databases, PubMed, Google, Google Scholar, and Research Gate. In addition to the relevancy of the title and abstracts, for strong conceptual and logical understanding, more recent studies were selected for inclusion based on the year of publication, which was between 2016 and 2021. Though, little older publications and some news agencies, government data reports were also cited to strengthen the background of the subject. All selected articles have been cited accordingly.

## Malnutrition: Now and Then

Nowadays, malnutrition is considered as a complex problem to solve and has been recognized as a world health crisis: a major risk factor for various medical illnesses. Thanks to the vision “Sustainable Development Goals (SDG) 2030, since 1990, there has been a 50% reduction in under-5 years” children deaths by malnutrition (22). To address the challenge of malnutrition, SDG aimed to reduce the under-5 mortality in all countries to at least as low as 25/1,000 live births by 2030 (23). WHO and United Nations International Children's Emergency Fund (UNICEF) integrated measurements are aimed to reduce the number of stunted children by 40% and wasting to <5% by 2025 (24). Although, we have made large-scale efforts on the lives of malnourished children in developing countries around the world, however, due to the exponentially raised population number, however, due to rising population, complexities in screening, and the association of malnutrition with other infections, current estimates predict that these measures are still insufficient to treat, control, and reduce the burden. More effective prevention and treatment of malnutrition are needed urgently.

## Underlying Causes of Malnutrition

Any disease, whether chronic or acute, is a significant risk factor of malnutrition in third world countries and could cause or exaggerate malnutrition in some respects. As aforementioned, malnutrition is considered as a complex problem to solve due to the contribution of several driving factors in its development, so there is no way to list out the factors that may contribute to malnutrition in the population (21). With this complex manifestation across the life courses in different ways, it has been found that the risk factors and co-occurrence of malnutrition are not only related to nutrient intake but also linked with the levels of water sanitation and hygiene and the influence of infections on nutritional status (25–28).

**Figure 1:**
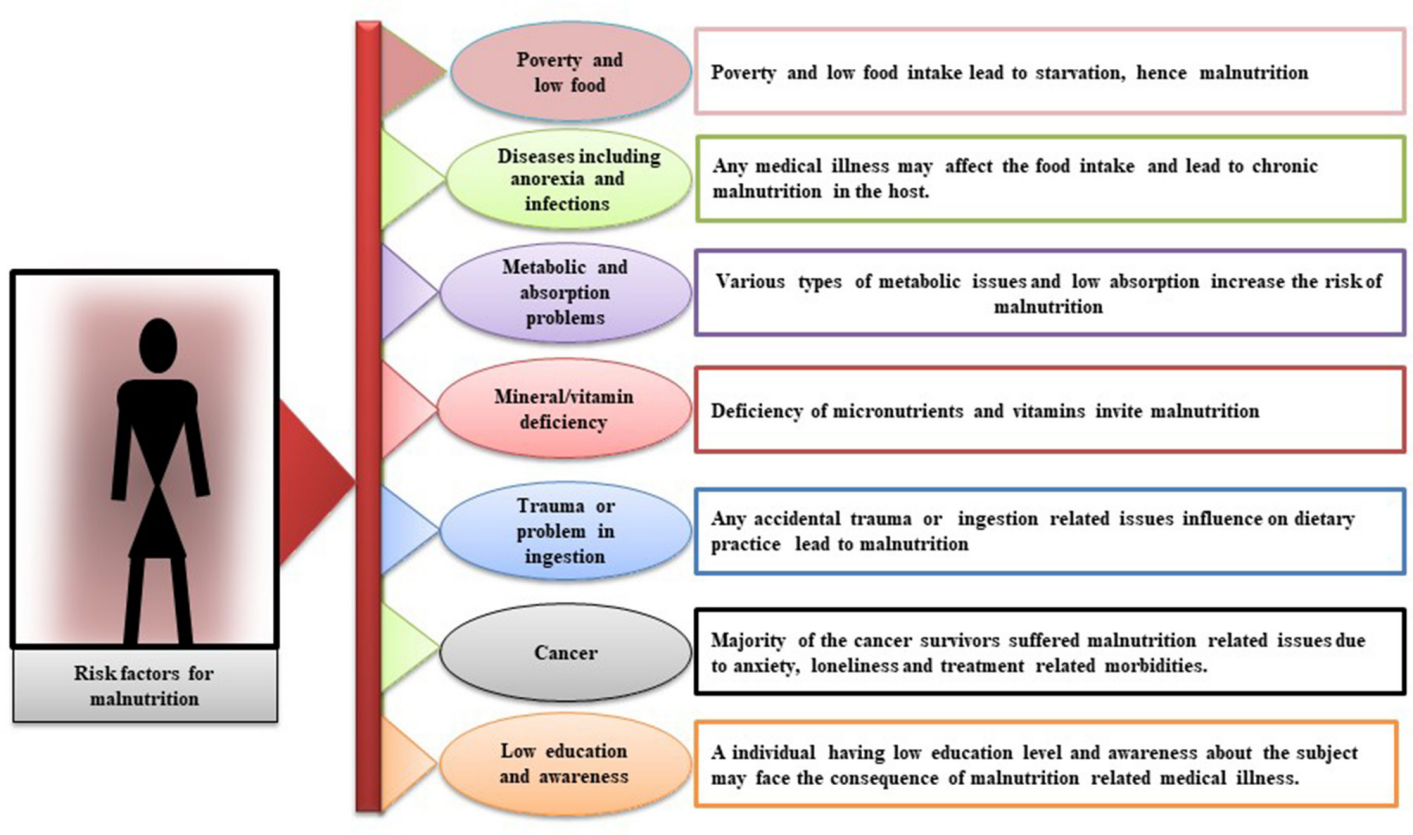
Diagrammatic description of risk factors that contribute directly or indirectly to the development of malnutrition. Leading facts are poverty and low food, diseases, such as anorexia and infections, metabolic and absorption problems, mineral/vitamin deficiency, trauma or problem in ingestion, cancer, and low education and awareness.

In elderly persons, malnutrition is a serious threat that leads to disability, institutionalization, and more exposure to illnesses. In these patients, the underlying cause of being malnourished is anorexia of aging: an individual's food intake decreases with age (29). Clinically, metabolism-related malnutrition might be a result of reduced assimilation of nutrients and malabsorption, any infection in the digestive system, trauma, or any type of inflammation (30–32). Different studies have revealed that there are several mediators, such as cytokines (interleukin 1, interleukin 6, and tumor necrosis factors alpha), glucocorticoid, and lack of insulin growth factor 1, whose catabolic effects have altered food intake (33–35). Poor dentistry is also the result of a number of factors, especially in geriatric patients, such as dementia and immobilization (36–38). Other factors, such as loss of appetite, altered taste, and nausea, can reduce food intake and thus malnutrition (39, 40). Socioeconomic factors, such as isolation, poverty, and poor living conditions, are involved in the progression of malnutrition (17, 41). Malnutrition commonly occurs in elderly patients and is highly prevalent in patients with malignant/severe chronic liver disease, heart or kidney disease, HIV/AIDS, chronic obstructive pulmonary disease, impaired intestinal flora and cystic fibrosis, respiratory tuberculosis, and other diseases (42–51). Various studies have shown that patients tend to receive less nutritional care due to the inexperience of hospital staff (52). In addition, malnutrition is also associated with loss of appetite, poor diet, physical disability, depression, and loneliness (53, 54). Diseases are one of the major factors of malnutrition development and the risk increases with the severity of the diseases and it makes it exceedingly difficult to analyze the effect of malnutrition on prognosis alone. This can only be done in well-characterized settings, where analysis can be stratified according to the severity of the disease. A diagrammatic description of all direct or indirect factors that mediate in bringing the malnutrition has been shown in Figure 1.

## Protein Energy Malnutrition and Infection

Protein energy malnutrition is a range of pathological conditions arising out of coincident of lack of protein and energy in varying proportions, most frequently seen in infants, and young children and usually associated with infections (55). It is understandable that the body needs extra energy to activate and replenish the body's immune system in response to an infection. In nutrition sciences, this association of energy expenditure during infection can be expressed in the form of PEM, which provides an outlook to understand the relationship between malnutrition and infection (56). WHO has identified PEM as the most lethal form of malnutrition as it affects the most vulnerable segment of the population: children under the age of 5 (57). Through the years of research on the association of malnutrition and infection, it has been well-established that, in malnourished individuals, the immune capacity of energy expenditure is further impaired, and therefore the host becomes susceptible to infection and determines the outcome of infection (58).

**Figure 2:**
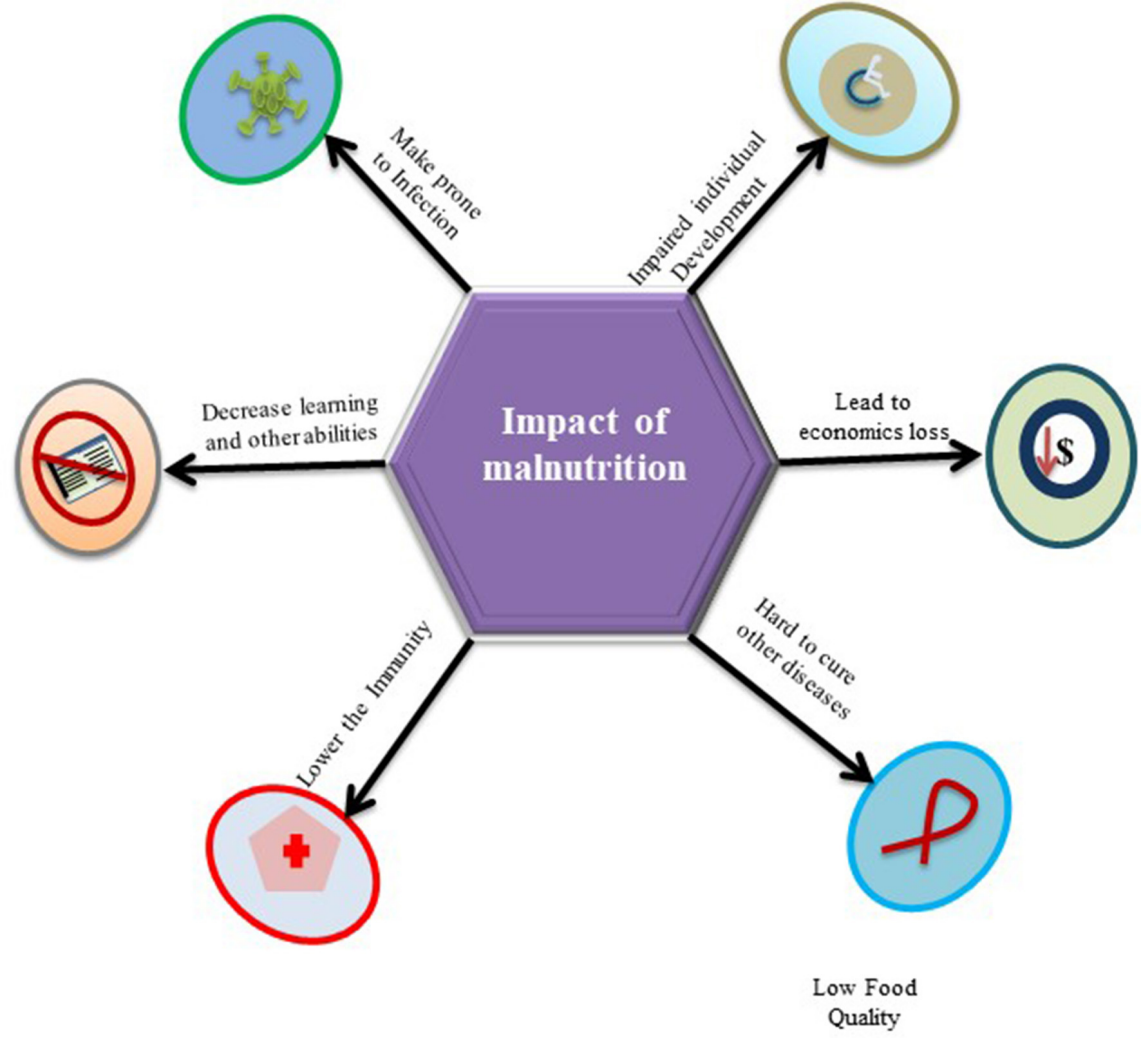
The figurative presentation of impose-impact in the form of low immunity and prone to infections with other related outcomes by malnutrition on an individuals' life.

Although, deficiencies in non-nutrients, such as vitamins, amino acids, iron, and even micronutrients, can affect the normal functioning of the body, however, PEM is considered to be associated with delayed recovery from disease, poorer quality of life, and increased risk of morbidity and mortality (59). It makes the body vulnerable to major human infectious diseases, especially in children in low-income countries (22). Overviewing the published data, it has been found that around half of the 10.8 million annual deaths of children under the age of 5 by malnutrition in less developed countries are under the deadly co-existence of PEM and infections (60). Hence, PEM determines the susceptibility of infection in the body and energy of the individual, which further leads to a loss of work efficiency at the community level and catalyzes the alarming spiral of infection, disease, and blatant poverty in society. Figure 2 demonstrates impose-impact of malnutrition on an individuals' life, such as impaired growth, economic loss, hard to recover from other medical illnesses, low immunity, and prone to infections.

## Malnutrition Increase Risk of Infection

**Figure 3:**
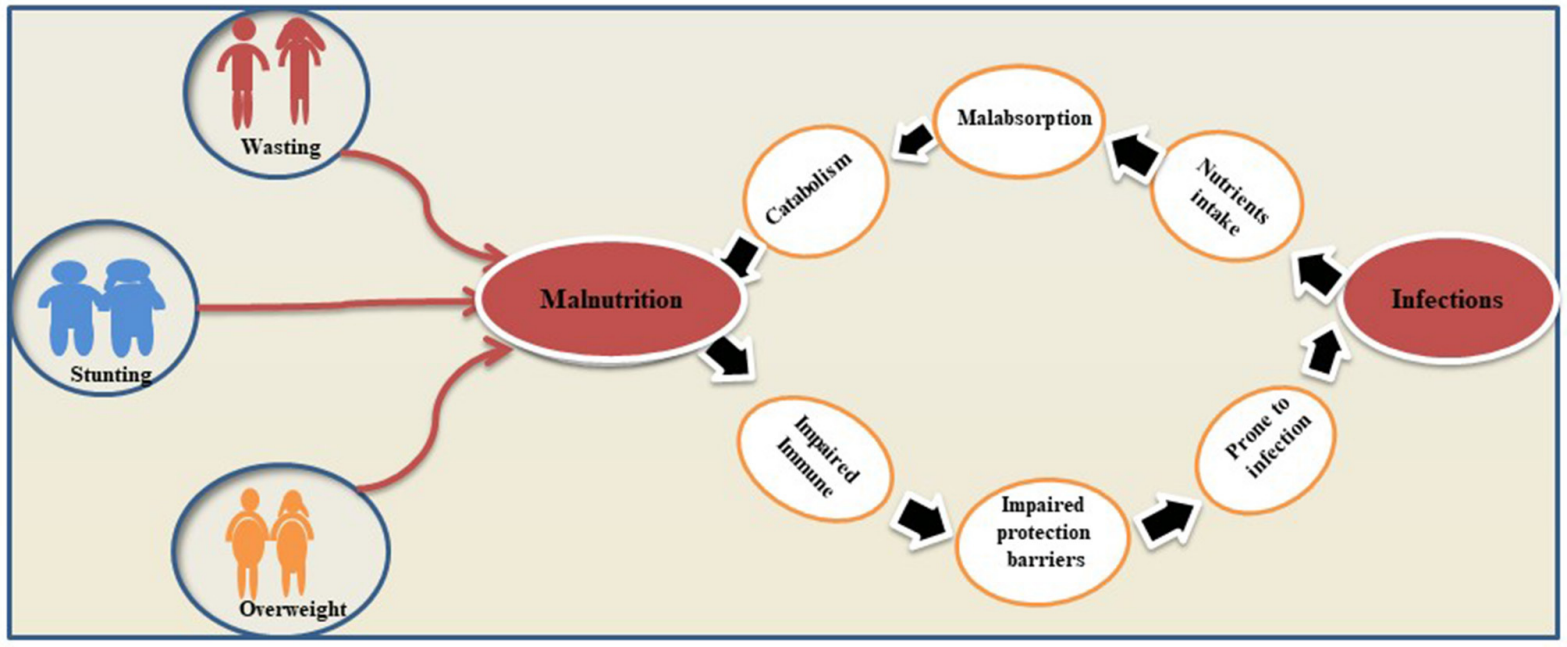
Bidirectional associations between malnutrition and infections. The figure gives an explanation on how malnutrition and infections reinforce each other's and lead to maximum health damage of host.

A vicious relationship between malnutrition and infectious diseases has been long established. Malnutrition causes an exponential decrease in the chances of survival per exposure to any fatal infectious disease while non-fatal and subclinical infections may impair the process of growth (61). Published research revealed that malnourished children have a clear excess risk of infectious morbidity and mortality (62). Concurrently, the latest estimates show that about 50% of 10.6 million under 5 child death per year globally has been ascribed to the five infectious diseases (pneumonia, diarrhea, malaria, measles, and AIDS) (25, 63). Malnutrition impaired the integrity of the gastrointestinal mucosa of patients leading to reduced gastric acid secretion and increased susceptibility to some pathogens (64, 65). Besides the direct organ-specific effects of infection (e.g., intestinal loss of nutrients during diarrhea), there are other illnesses, such as metabolic and immune systems, which are the result of this association (66). It has been well-established that HIV infection and malnutrition are inter-linked by the complex interplay of different etiological factors, which contribute to the progression of the disease and increase the risk of mortality. Hence, addressing the nutrition right from the time of HIV diagnosis is a good strategy. This reduction of immune cells, known as nutritionally acquired immunodeficiency syndrome, plays a key role in the body's susceptibility to any infection (67). Severely malnourished patients have lacked a strong immunological response and show weak acquired immunity and innate host defense mechanisms. These patients are more susceptible to infection factors than the patients with HIV/AIDS and their opportunistic pathogens (68). A remarkable example of malnutrition and infection was seen after the Second World War when thousands of patients with AIDS and malnourished children were repeatedly diagnosed with opportunistic fungal pneumonia with other illnesses (69). Similarly, Noma is another deadly opportunistic infection. Noma is a result of complex interactions of different factors, such as poverty, malnutrition, compromised immune system, and poor oral hygiene, typically occurring in sub-Saharan Africa in children under 4 years of age (70). Severe malnutrition, PEM, promotes acute and chronic infections, further reduces food intake, increases metabolic requirements, and reduces anabolic nutrient losses by impairing linear growth in affected children. Additionally, the acute phase of PEM disrupts bone remodeling required for longitudinal growth (71). It has been pointed out that the association between malnutrition and growth retardation helps researchers to assess the nutritional status of individuals. Numerous studies have demonstrated a significant correlation between acute malnutrition and acute infections. It has been well-established by the years of research that one cannot separate infection and its risk factors as determinants of the whole malnutrition burden. These determinants of malnutrition and infections are significantly present in the under-5 years of age children in developing countries and adversely impact children's health and development (25, 72, 73). Overviewing the published literature, it has been concluded that the relationship between malnutrition and infection is considered bidirectional (74). Evidence of the inverse relationship between malnutrition and infection is also found where an infection alters the nutrients intake, absorption, secretion, catabolism, and consumption. Such a vicious cycle of infection and malnutrition appears commonly in nature (25). Figure 3 represents the bidirectional associations between malnutrition and infections.

## Malnutrition and Chronic Diseases

It is a need of time to add important nuance to establish facts and understandings about the complex associations of chronic diseases, such as diarrhea, respiratory infection, HIV, malaria, measles, and the most hateful disease cancer, with malnutrition for the specific therapeutic approaches to break these associations and treat the diseases.

The vicious cycle between malnutrition and diarrhea is being explored for decades of years. According to a statistic report, around 1.5 million children die every year of diarrhea (75). It has been understood that, as compared to better nourished children with diarrhea, malnourished children with diarrhea have a far higher risk of death (76, 77). Diarrhea is the most common morbidity in severe acute malnutrition children and needs to be addressed. Effective adherence to the management protocol of dehydration and prompt modification of therapeutic feed can bring satisfactory health outcomes.

Followed by diarrhea, respiratory infections are the leading causes of death in children under the age of 5 years (78). It is plausible to assess the relationship between respiratory infection and malnutrition as poor growth. The low immune response of the host and susceptibility to pathogens lead to mortality and morbidity among the children across the globe (79). Going through the literature, it has been found that the published articles that concluded that malnourished children are very susceptible to several respiratory infections as compared to the others (80–82). Malnutrition especially in children is the risk factor for many bacterial and viral infections. A study on hospitalized children with the severe respiratory syncytial virus (RSV) demonstrates that poor infant growth increases the risk for severe RSV infection and leads to prolonging hospitalized stay (83). Similarly, there are also findings about the frequency of co-morbidities related to measles, pneumonia, tuberculosis, and malaria in the children presenting with severe acute malnutrition (84, 85). In an observational study, it has been known that 22% of 104 severe acute malnourished children were diagnosed with tuberculosis while 3.8% were diagnosed with malaria and measles (86). The research published in the *American Journal of Clinical Nutrition*, with the objective to find whether undernutrition is an underlying cause of children deaths associated with diarrhea, pneumonia, malaria, and measles, reveals that overall 52.5% of all deaths in young children were attributable to undernutrition varying from 44.8% for death by measles and 60.7% for death because of diarrhea (87).

**Figure 4:**
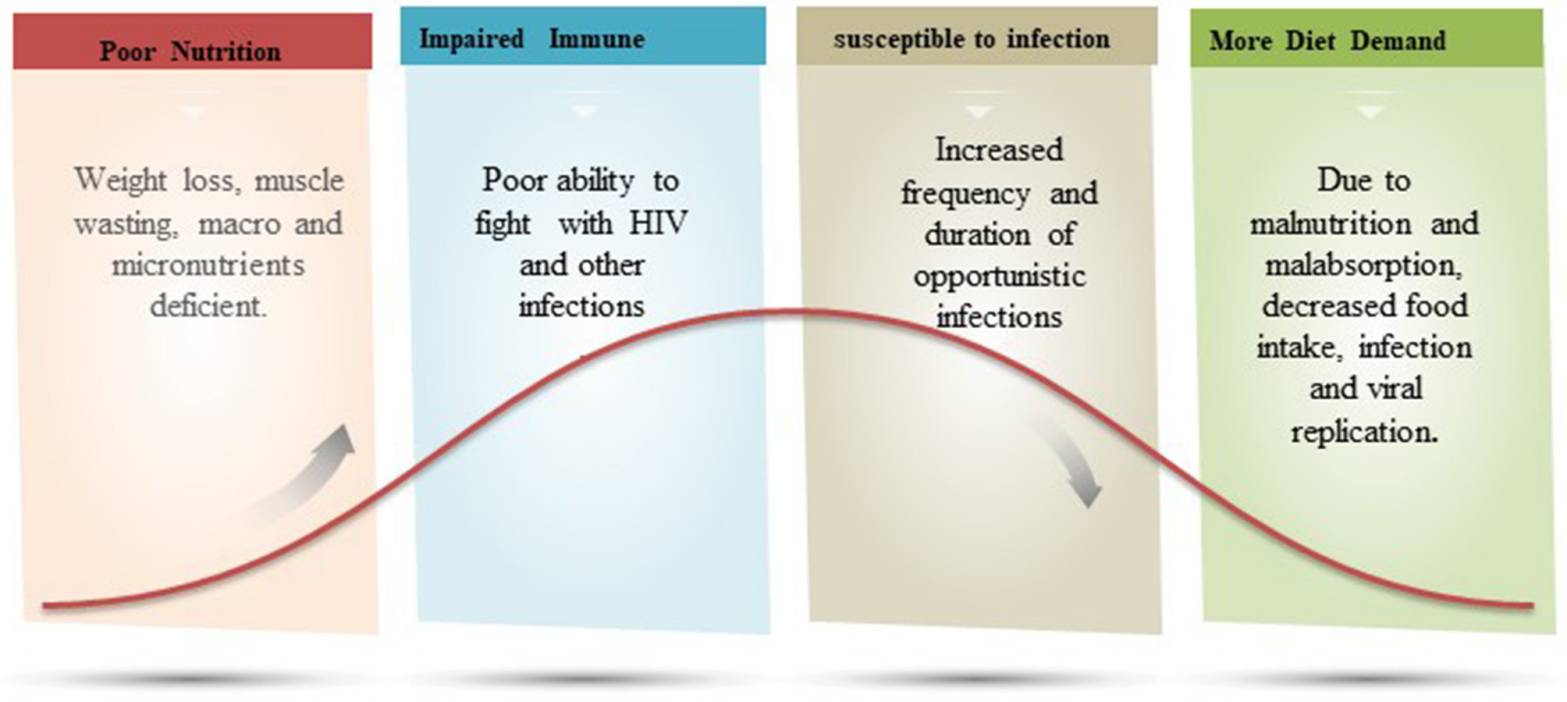
A figurative explanation on the relation of malnutrition and immune fighting ability against HIV infection. In a vicious cycle of malnutrition-HIV, the patient has poor immunity and increased frequency and duration of opportunistic infection.

Due to the desperate consequences of malnutrition, more than 1 million children die each year with the co-occurrence of HIV (88). The comorbidity rate of malnutrition and HIV makes HIV-infected children three times more likely to die than non-infected children (88, 89). Sub-Saharan Africa is the most affected region by the deadly association of malnutrition and HIV where 31.2% of stunting, 7.4% of wastage, and 5.2% of overweight children are reported (90). HIV infection in malnourished individuals is the result of several physiological and socioeconomic factors (91, 92). Muenchhoff confirmed strong associations with other comorbidities, such as diversification of the gut microbiota, epithelial malformations, chronic intestinal inflammation, and immune activation in malnourished HIV-infected children, which further lead to advances in disease pathogenesis (88). According to a recent study, it has been observed that micronutrient deficiency, such as selenium and vitamin C, determines the mycobacterial progression in HIV-positive patients, thus an imbalance of these micronutrients can be considered as an important risk factor (93). Furthermore, it has been found that in HIV-positive cases, metabolic problems, such as high lipids, can lead to increased long-term risk of atherosclerosis and cardiovascular abnormalities in malnourished patients (94). Figure 4 represents the vicious cycle of malnutrition and HIV.

Despite the immense volume of laboratory and field research, there is still an adequate gap in knowledge about the subject and in defining the relationship between malnutrition and chronic diseases. To deal with this worldwide health crisis, diverse minds of researchers are continuously exploring the deadly associations of malnutrition and chronic diseases for the vision of a “world free of malnutrition.”

Here, we are illuminating the current understanding of the interactions between malnutrition and cancer. As secondary data, this is an objective study to summarizing the literature and providing an update on the association of malnutrition and said disease.

## Malnutrition and Cancer

Malnutrition is exceedingly common in cancer patients. Cancer-associated malnutrition is a multifactorial syndrome characterized by persistent skeletal muscle loss due to different combinations of reduced dietary intake and metabolic changes (42). This association might express at the time of diagnosis or develop later and as the treatment and disease progressed, the situation became worse. The nutritional status of cancer patients who underwent treatment plays a vital role in promoting multimodality cancer care (95).

Recently, studies demonstrate that many European hospitals have 30–60% of cancer patients at risk of malnutrition who receive nutrition through other means, such as oral supplements, enteral nutrition, or parenteral nutrition (96, 97). Another European study showed that 40% of cancer-related severe malnutrition was misclassified by doctors, resulting in patients losing nutritional interventions (43). It has been also noted that damage of cancer-related malnutrition went uncontrolled when patients and their relatives underestimate it after the doctor's diagnosis (98–100).

**Figure 5:**
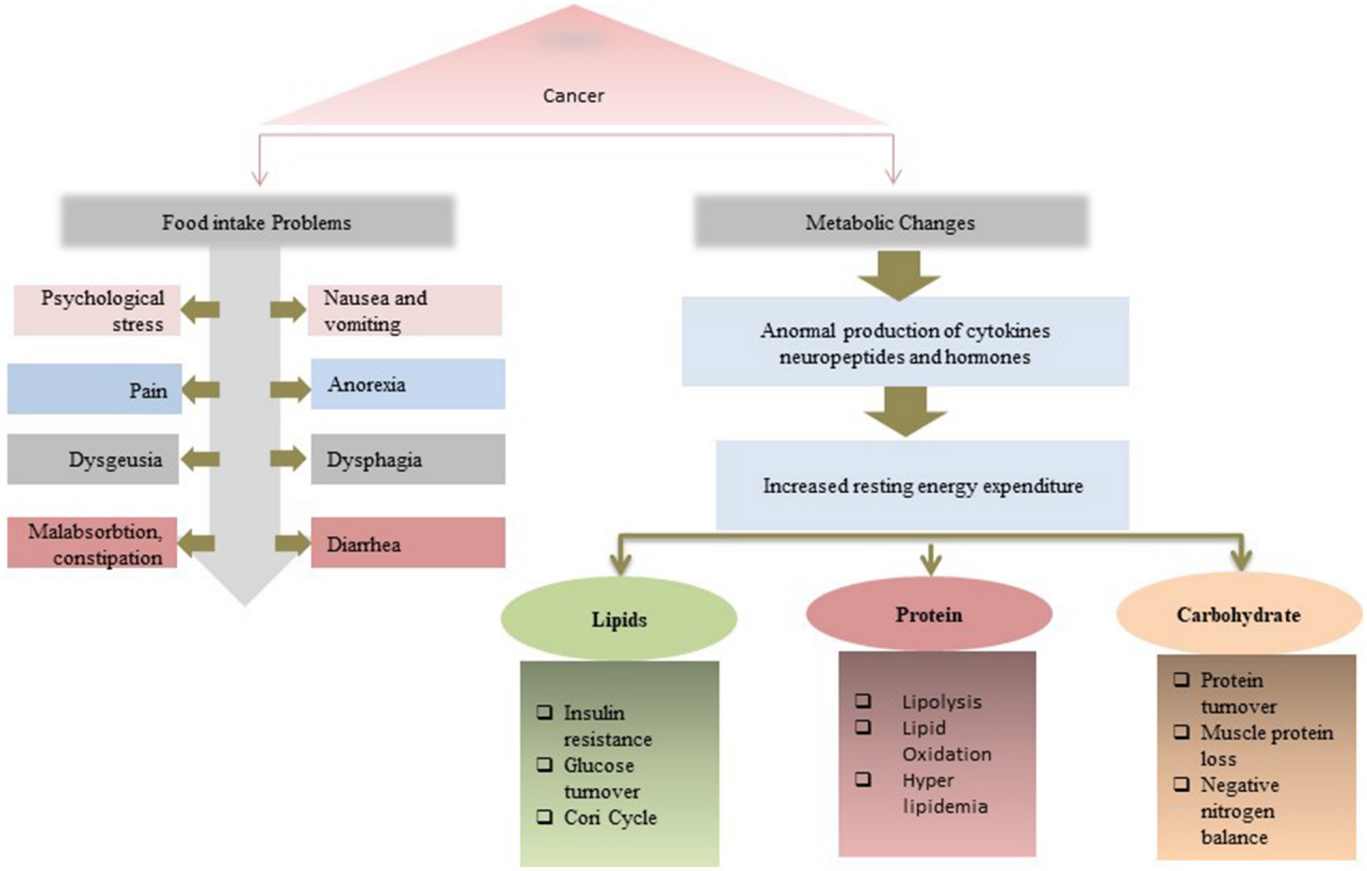
Multi ways associations and outcomes of the relation between malnutrition and cancer. A malnutrition-cancer patient faces issues both in food intake and metabolic changes that lead to the reduction in treatment efficiency.

The increasing frequency of malnutrition has become an emerging health problem of cancer survivors as it decreases the efficacy of homeopathy treatments and prolongs hospital stay especially in the citizen of the United States of America (101, 102). According to a follow-up study, it has been obtained that malnourished patients with cancer are at about 2- to 5-fold greater risk of death (103). The development of cancer-related malnutrition is the result of several factors, which are considerably different from simple starvation, such as mental health issues, poor food intake, dysfunction of the gastrointestinal tract, increase in the energy need, low physical activity, and altered metabolism (42, 43, 104). Tumor-derived cytokines also mediate the disruption of metabolism, suppress the appetite, enhance muscle wasting, depression, fatigue, and subsequently, lead to impaired physical activity and anorexia (43, 105). Mentioned conditions may worsen the prognosis and lead to poor quality of life, prolonged hospital stay, tolerance to treatment, and therapeutic efficacy that lead to the end of life (106, 107). Additionally, cancer patients often report poor appetite along with reduced food enjoyment putting them at risk for malnutrition. This reduction in food intake may increase due to the site of the tumor. For example, oral or esophagus cancer may halt the eating and support the malnutrition onset (107, 108). On the other hand, the use of steroid drugs and hormone therapy has been considered as a standard of care for a different advanced type of cancer, however, there are findings that reveal that these steroid or hormone intake may contribute to the development of malnutrition (109, 110). Furthermore, unlikely other disease-related malnutrition, cancer-related malnutrition, are thought to be different from those that lack nutrients in the absence of underlying diseases (i.e., hunger and anorexia nervosa). It has been observed that metabolic changes in cancer patients, such as inflammation, increased catabolic rate, and ineffective cyclic anabolic resistance, achieved through a tumor or cancer treatment may invite malnutrition in the patient (111, 112). Apart from other clinical aspects in cancer-related malnutrition, the lack of adequate knowledge of nutritional intervention for cancer patients in caregivers is also a significant contribution in cancer-related malnutrition loss. A recent study demonstrates that almost half of the hospital oncologists involved in the survey report ascribe the underestimation of nutritional treatment to the insufficient training of healthcare professionals (95). In conclusion, to end up this deadly link of malnutrition and cancer, medical science is an urgent need to introduce advanced, state-of-the-art diagnostics, and care mechanisms and emphasizes the concern institutes to spread and raised the prevention and awareness literature to the public. Figure 5 gives the multi ways associations and outcomes of the relation between malnutrition and cancer.

## Prevention and Awareness of Malnutrition: Public Health Nursing

Exceedingly damage by malnutrition-related co-morbidities demands the development of the specific protocol and practical recommendations rather than official meetings for the prevention and awareness about this global health crisis.

Multidisciplinary approaches are needed for the long-term nutritional support and benefits of geriatric malnutrition prevention, detection, and treatment. These approaches must explain acceptable different screening/assessment models and involve healthcare professionals (113). To examine and prevent the PEM especially in growing children from developing countries, there is a critical need of employing electrical health records and other mobiles systems. Adaptation of health information technology is a single advanced way forward of achieving better health outcomes across the globe (114). Malnutrition manifests its worse outcome in hospitalized malnutrition form and needs effective strategies to promote the awareness and overcome the burden. The nutrition committee of the French pediatric society (ePINUT) has endorsed an awareness strategy to lessen the burden and released its recommendations about the prevention and promotion of malnutrition education awareness in patient relatives, healthcare staff, and other caregivers (115). The vicious association of malnutrition cancer imposes significant health and economic loss. Cancer-related malnutrition is a globally rising health concern and appeals to add novel nuance to break this association. According to a current survey report, there is a lack of collaboration between oncologists and clinical nutritionists, which is the first obstacle to overcome. We are in urgent need to start internationally accepted educational intersociety initiatives, aimed to strengthen oncologist knowledge to improve nutritional support management and identify and remove the barriers to delivering optimal nutrition care in cancer survivors (116, 117). To lessen the burden and to the way of the world free of malnutrition, current understandings of knowledge about the subject deduced the following key steps to start a war against malnutrition. (1) Awareness about all forms of malnutrition must be raised: frontline staff who provide care must understand the importance of nutrition to basic patient care and consequences to patients if they neglected; (2) there is a critical need to develop worldwide accepted state of the art screening and assessments methods; (3) malnourished individual or those at risk must be on the right care pathway and received the right treatments; (4) frontline staff in all care settings must receive appropriate training on the importance of good nutritional care to speed up the recovery; and (5) world organizations must development management structure in place to ensure best practice especially in high prevalence parts of the world: Sub-Saharan Africa.

## Conclusion

The literature revealed the need for important implications of nutritional intervention drives for elderly and child survival programs for developing countries to ensure the access of children who fight against chronic diseases. In conclusion, to fill the gaps in prioritizing standardized measurements and reporting of malnutrition status worldwide, it is imperative to enhance an in-depth understanding of the development causes and co-existence of malnutrition with other illnesses. To overcome malnutrition, it also demands to re-make and re-define the future by giving comprehensive but achievable and sustainable programs to alleviate poverty and combat the rampant infectious diseases and other nutrition-related health problems. To improve health outcomes especially in hospitalized cancer patients, it is crucially important to knowledge the caregiver healthcare staff for early interventions of supplying nutritional support to delay or prevent the onset of malnutrition.

## Author Contributions

YF, YL, TJ, and QY reviewed the literature and prepared the manuscript draft and the figure. EJ and JZ made the final editing and offered his expert suggestions and insights in preparing this work. All authors have read and agreed to the published version of the manuscript.

## Funding

This review was supported by the National Natural Science Foundation of China (No. 81900375) and the Henan Provincial Science and Technology Research Project (No. 212102310147).

## Conflict of Interest

The authors declare that the research was conducted in the absence of any commercial or financial relationships that could be construed as a potential conflict of interest.

## Publisher's Note

All claims expressed in this article are solely those of the authors and do not necessarily represent those of their affiliated organizations, or those of the publisher, the editors and the reviewers. Any product that may be evaluated in this article, or claim that may be made by its manufacturer, is not guaranteed or endorsed by the publisher.
